# Outcomes of immunocompromised and non-immunocompromised patients in the ICU diverge late in the pandemic

**DOI:** 10.1371/journal.pone.0357181

**Published:** 2026-09-09

**Authors:** Shreya Battu, Andrew R. Moore, Katie Lebold, Ana Pacheco-Navarro, Shaun Pienkos, Christian O’Donnell, Pablo Sanchez, Caitlin Parmer-Chow, Jonasel Roque, Tara Ramaswamy, Jennifer G. Wilson, Joseph E. Levitt, William Collins, Angela J. Rogers

**Affiliations:** 1 Division of Pulmonary, Allergy and Critical Care Medicine, Stanford University School of Medicine, Stanford, California, United States of America; 2 Division of Pulmonary, Allergy and Critical Care Medicine, Oregon Health & Sciences University, Portland, Oregon, United States of America; 3 Department of Medicine, Stanford University School of Medicine, Stanford, California, United States of America; 4 Department of Medicine, University of California San Francisco, San Francisco, California, United States of America; 5 Department of Anesthesiology, Stanford University, Stanford, California, United States of America; 6 Department of Emergency Medicine, Stanford University, Stanford, California, United States of America; 7 Division of Hospital Medicine, Stanford University, Stanford, California, United States of America; University of Arkansas for Medical Sciences, UNITED STATES OF AMERICA

## Abstract

**Rationale:**

The impact of immunocompromising conditions on outcomes in critically ill patients with COVID-19 remains poorly characterized. We hypothesized that disparities in COVID-19 outcomes in immunocompromised patients may differ by viral strain and time course of the pandemic.

**Methods:**

We retrospectively reviewed SARS-CoV-2 RT-PCR positive patients admitted to intensive care (ICU) at Stanford from March 2020 to July 2022 using electronic medical records. Immunocompromised status included solid organ transplant; hematologic malignancy/transplant; solid cancer with metastases and recent chemotherapy; HIV/AIDS; and connective tissue disease on immunosuppression. SARS-CoV-2 strain periods were classified as pre-Delta (3/2020–8/2021), Delta (9/2021-12/2021), and Omicron (1/2022–7/2022). We assessed 90-day survival differences between immunocompromised and non-immunocompromised patients by strain using Cox-proportional hazards, adjusted for age, sex, and APACHE II score.

**Results:**

A total of 791 patients were included, 450 from the pre-Delta period, 114 from the Delta period, and 227 from the Omicron period. We compared 633 non-immunocompromised patients with 158 immunocompromised patients (77 during the pre-Delta, 22 Delta, and 59 Omicron periods). Patients admitted during the Omicron period were more likely to be immunocompromised (26%) compared to pre-Delta (17%) or Delta periods (19%; p = 0.008). Immunocompromise was associated with worse outcomes in the entire cohort after adjustment (HR 1.68, 95% CI 1.2–2.3, p < 0.001). However, when stratified by strain period, immunocompromised status was only significantly associated with higher 90-day mortality in patients from the Omicron period (42% vs 17%, HR 2.9, CI% 1.7–5.0, p < 0.001). Differences in mortality between immunocompromised and immunocompetent patients were not statistically different in either the pre-Delta (34% vs. 23%, HR 1.4, 95% CI 0.9–2.1, p = 0.15) or Delta periods (32% vs. 28% HR 1.0, 95% CI 0.4–2.5, p = 0.92). Notably, survival was significantly improved during the Omicron period compared to earlier strain periods in immunocompetent patients (HR 0.6, 95% CI 0.4–0.9, p = 0.02) but not for immunocompromised patients (HR 1.4, 95% CI 0.80–2.44, p = 0.24).

**Conclusion:**

In this retrospective cohort analysis of patients admitted to the ICU due to SARS-CoV-2 infection, we found that lower survival among immunocompromised patients was due to higher mortality during the Omicron period, with similar mortality between immunocompetent and immunocompromised patients during other periods.

## Introduction

The COVID-19 pandemic left an indelible mark on the global landscape, claiming millions of lives worldwide. In the pre-COVID era, common respiratory viruses, such as influenza and respiratory syncytial virus (RSV), were associated with more severe outcomes in immunocompromised patients. For instance, a comprehensive study by Collins *et.al* (2020) showed that immunocompromised patients with influenza had an increased risk of dying relative to immunocompetent patients hospitalized with influenza [1]. Additionally, Dumas *et.al* found that 31% of immunocompromised patients admitted to the intensive care unit (ICU) with a respiratory virus ultimately died in the ICU [2]. Whether COVID-19 similarly led to more severe disease in immunocompromised patients remains uncertain.

Several studies early in the COVID pandemic reported that outcomes did not differ based on the presence of an immunocompromising condition [3,4], whereas others reported disparate outcomes only in certain subsets of immunocompromised patients [5–7]. More recent studies – especially from the post-vaccination era – have consistently demonstrated that immunocompromised individuals experience worse outcomes from SARS-CoV-2 infection compared to the general population.

Recent systematic reviews and cohort analyses have demonstrated that immunocompromised individuals remain at elevated risk for breakthrough infections, hospitalization, and death, even as treatment strategies and vaccination coverage have improved [8–12]. Elevated morbidity and mortality have been reported across a range of immunocompromised subgroups, including individuals with cancer, autoimmune or inflammatory conditions, and particularly those who have undergone lung transplantation [3,4,6,8]. The disparate findings between these more recent studies and the mixed findings from early in the pandemic likely reflect the complex relationship between immunocompromised states, evolving host immunity from vaccination or prior infection, and COVID-19 strain variation during different periods of the pandemic.

In the general population, differences in outcomes between the pre-Delta, Delta, and Omicron eras are well documented. Studies have highlighted significantly lower rates of hospitalizations and mortality in the Omicron variant compared to Alpha and Delta strains [13] and a higher risk of hospitalization among patients with the Delta variant than Alpha [14]. To better understand the relative impact of strain variation and evolving host immunity on outcomes in immunocompromised patients throughout the pandemic, we assessed the association between immunocompromised status and mortality across a broad period of the pandemic, inclusive of the three predominant COVID-19 viral strain periods.

## Methods

### Patient population and data abstraction

We performed a retrospective review of sequential patients with acute SARS-CoV-2 infection who required ICU admission in the Stanford Health Care System from March 2020 to July 2022. Stanford Health Care includes both an academic tertiary care center ICU and an ICU at a community affiliate hospital. All SARS-CoV-2 cases were confirmed by reverse polymerase chain reaction (RT-PCR) on a nasopharyngeal swab specimen. Baseline clinical and demographic characteristics, along with vitals and laboratory data, were abstracted through automated review by the STAnford Research Repository (STARR), a clinical informatic tool that allows for extraction of electronic health records for research purposes. COVID-19 strain periods were assigned based on local epidemiologic and institutional genomic surveillance data demonstrating distinct periods of variant predominance within the Stanford Health Care population, rather than patient-level viral sequencing, as this data was not available. The pre-Delta period spanned from March 2020 to August 31, 2021; the Delta period from September 1, 2021 to December 31, 2021; and the Omicron period from January 1, 2022 to July 2022 [15–17]. These cutoffs were selected based on internal epidemiologic data demonstrating sequential dominance of each variant within our healthcare system, consistent with broader regional and national trends [15–17]. APACHE II scores were calculated using date of ICU admission, excluding points for immunocompromised status to avoid biasing analyses of this primary variable of interest. Shock was defined by the requirement for vasopressor support at time of ICU admission.

Immunocompromised status was manually abstracted from electronic medical records (EMR, Epic Systems). Trained reviewers performed detailed chart review to capture relevant immunocompromising conditions, rather than relying solely on diagnostic codes or automated flags. This manual phenotyping allowed for classification by incorporating provider documentation, medication history, and consultant notes. Immunocompromised status was defined by the presence of a solid organ transplant (SOT); bone marrow transplant or active hematologic malignancy; solid cancer with metastases and received chemotherapy within the six months prior to ICU admission; HIV/AIDS with baseline CD4 counts <200; and connective tissue disease (CTD).

All data were accessed for research purposes between May 1, 2023 and December 10, 2025 and all data collection was performed in accordance with Stanford IRB 56374. During data abstraction and manual chart review, study personnel had access to identifiable participant information; however, all analyses were conducted on de-identified datasets. All procedures were followed in accordance with the Helsinki Declaration. Reporting also adhered to the STROBE (Strengthening the Reporting of Observational Studies in Epidemiology) guidelines checklist for observational studies [Cuschieri S, 2019].

### Statistical analysis

We evaluated differences in the proportion of immunocompromised patients admitted to the ICU between predominant strain periods using Fisher’s exact test (i.e., pre-delta vs other, delta vs other, and omicron vs other). We then performed logistic regression to evaluate the association of strain with immunocompromised status after adjustment for age and sex.

To test whether immunocompromised status was associated with outcomes, we performed Cox proportional hazards modeling 90-day survival in immunocompromised patients versus non-immunocompromised patients, adjusting for age, sex, and baseline APACHE II score. To better understand the roles of host immunity and COVID-19 strain, we first evaluated outcomes across all patients and then evaluated differences by strain, first as a joint model and then by a strain-specific model stratified by immunocompromised status.

To evaluate the potential for confounding by differential vaccination status between strain periods on outcomes in immunocompromised and immunocompetent patients, we performed a sensitivity analysis including only patients with a documented full primary vaccination series (1 dose of the J&J vaccine or 2 doses of the Moderna or Pfizer vaccines) at least 14 days prior to ICU admission. However, we were unable to compare outcomes between vaccinated and unvaccinated patients because the EMR had a high rate of false negative vaccine status (i.e., patients who were in fact vaccinated but had no record of vaccination in the EMR).

As the COVID-19 pandemic progressed, we observed an increase in patients admitted with but not because of COVID-19 (i.e., “incidental COVID”). As a result, patients with later COVID-19 strains were more likely to be admitted to the ICU for etiologies other than COVID-19. To ensure that our results were not driven by this confounding factor, we performed an additional sensitivity analysis limited to patients who were on high flow nasal cannula, non-invasive positive pressure ventilation, or mechanical ventilation at time of ICU admission. To assess residual confounding by comorbidity burden, we performed an additional sensitivity analysis repeating the primary Cox proportional-hazards models with further adjustment for the Charlson Comorbidity Index.

All statistical analyses were performed using R Statistical Software (v4.2.2). Packages used for analysis included survival (v3.5-5), tidyverse(v2.0.0), and survminer(v0.4.9). Statistical tests were two-sided, and a p-value of less than 0.05 was considered significant.

## Results

**Fig 1:**
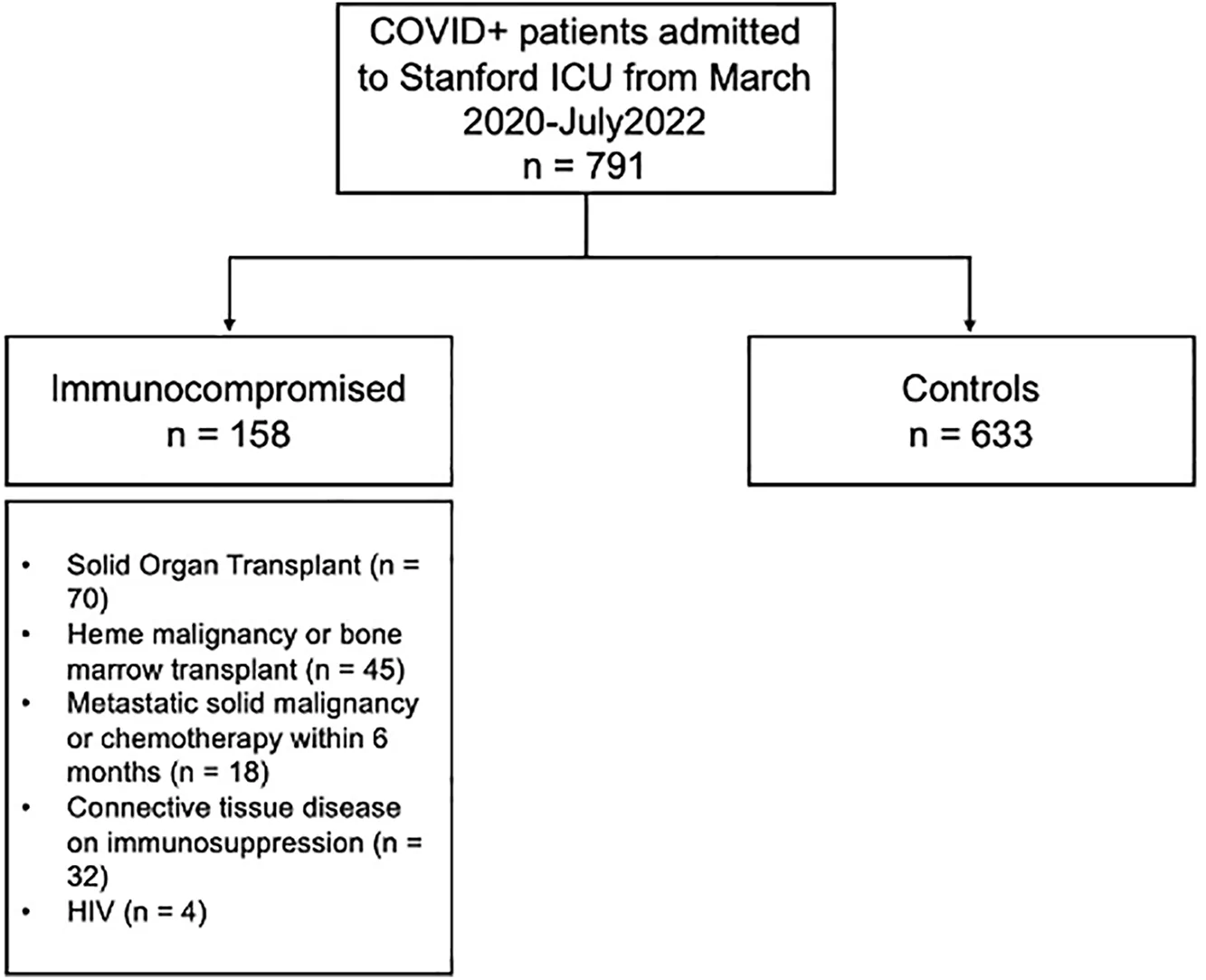
STROBE flow diagram of cohort selection.

Of the 791 adult patients with COVID-19 admitted to the ICU between March 2020 and July 2022, 450 were during the pre-Delta, 114 during Delta, and 227 during the Omicron period. This cohort included 158 (20%) immunocompromised patients and 633 (80%) non-immunocompromised patients (Fig 1). Among the 158 immunocompromised patients, 77 were in the pre-Delta, 22 Delta, and 59 Omicron periods. Among the immunocompromised patients, 70 (44%) had a solid organ transplant, 45 (28%) had an active hematologic malignancy or bone marrow transplant, and 32 (20%) had connective tissue disease on active immunosuppression. Demographics, including age, gender, and ethnicity, were similar across all strains and immunocompromised status (Table 1, Supplemental Table 1 in S1 File). While baseline APACHE II score was slightly higher in immunocompromised patients (18 vs 16, p < .0001, excluding points for immunocompromised status), rates of shock and mechanical ventilation were the same at time of ICU admission. Utilization of major COVID-19 directed therapies was prevalent across all strain periods**.**

**Table 1:** Baseline demographics and clinical characteristics of ICU patients with SARS-CoV-2 infection by immunocompromised status. Values are presented as counts with percentages or medians with interquartile ranges, as appropriate.

	Immunocompromised	Immunocompetent	All	p-value
**Total**	158	633	791	
Age (median, IQR)	61 [52, 70]	61 [44, 74]	61 [46, 73]	
Gender (n, % female)	74 (47%)	266 (41.8%)	340 (43%)	
**Race/Ethnicity**	**–**	**–**	**–**	
Asian/Pacific Islander	25 (15.9%)	106 (16.7%)	131 (16.6%)	
Black/Native American	13 (8.2%)	34 (5.3%)	47 (5.9%)	
White	44 (28%)	176 (28%)	220 (28%)	
Hispanic/Latino	58 (37%)	244 (39%)	302 (38%)	
Other/Unknown	18 (11.4%)	73 (11.5%)	91 (11.5%)	
**Strain**	**–**	**–**	**–**	
Alpha	77 (49%)	373 (59%)	450 (57%)	
Delta	22 (14%)	92 (15%)	114 (14%)	
Omicron	59 (37%)	168 (27%)	227 (29%)	
**Markers of Severity [n (%)]**	**–**	**–**	**–**	**–**
Shock	67 (42%)	271 (43%)	338 (43%)	**p = 1.00**
APACHE (median, IQR)	18 [14, 23]	16 [11, 23]	16 [11, 23]	p < 0.001
Intubated (n, %)	53 (34%)	219 (35%)	272 (34%)	p = 0.85
**Vent Free Days (median/IQR)**	23 [8, 28]	27 [9, 28]	27 [9,28]	p < 0.001
**90-Day Mortality**	58 (37%)	142 (22%)	200 (25%)	p < 0.001

### Proportion of immunocompromised patients varied by SARS-CoV-2 strain

The proportion of immunocompromised ICU patients increased across SARS-CoV-2 strain periods, from 17% in pre-Delta to 19% during Delta, and 26% during Omicron. In comparison to the pre-Delta and Delta periods, ICU patients admitted during the Omicron period had significantly higher odds of being immunocompromised (OR 1.69, 95% CI 1.15–2.49, p = 0.008) after adjustment for age and sex. In contrast, there was no difference in the proportion of immunocompromised patients admitted during the pre-Delta and Delta strain periods (p = 0.6, Supplemental Table 2 in S1 File).

### Immunocompromised status was associated with worse outcomes from COVID-19 critical illness only during the Omicron strain period

**Fig 2:**
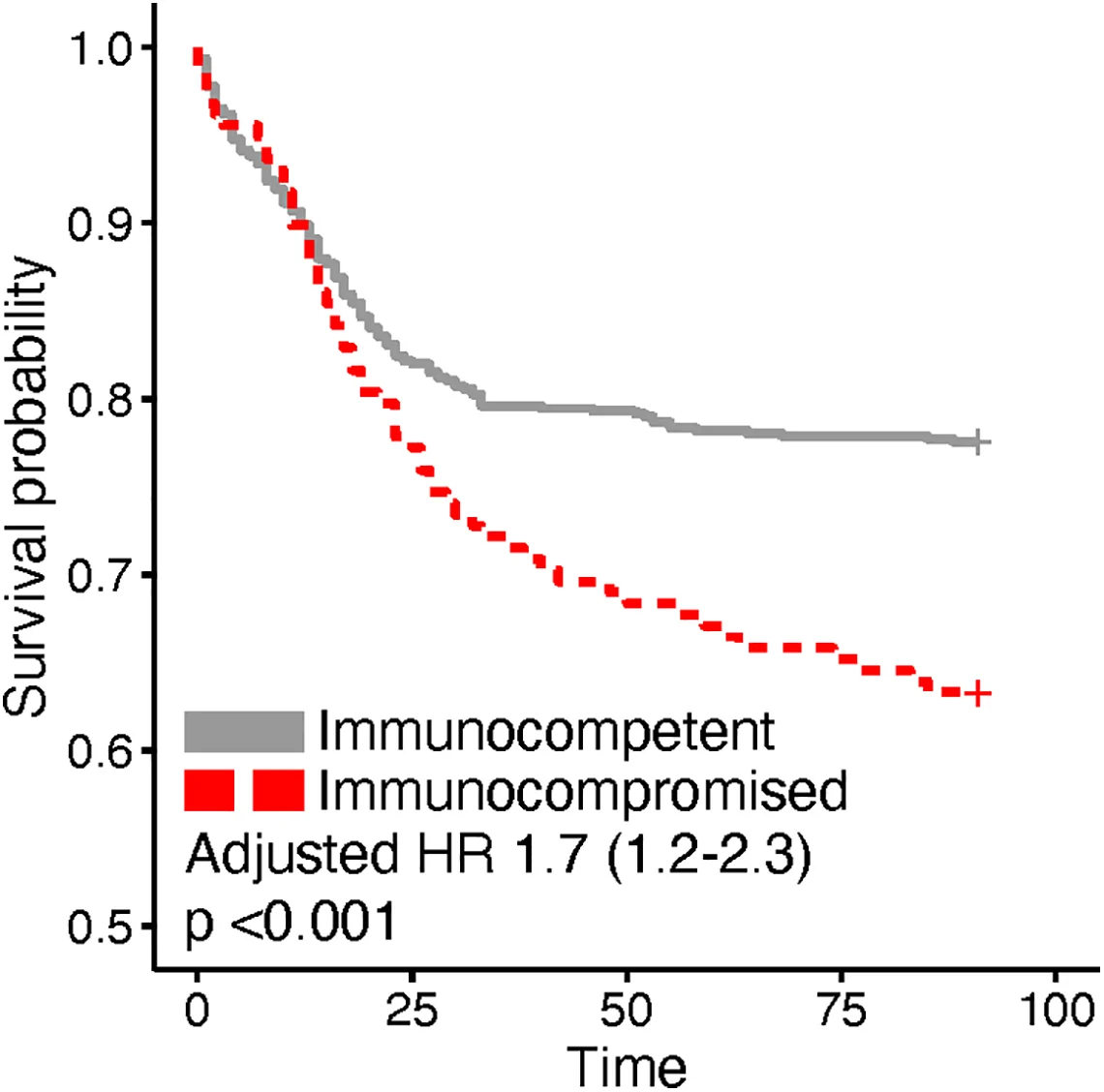
Kaplan-Meier survival curves evaluating 90-day survival in immunocompromised versus immunocompetent patients admitted to the ICU with SARS-CoV-2 infection. Hazard ratio (HR) represents cox-proportion hazards ratio for 90-day survival in immunocompromised patients relative to immunocompetent adjusted for age, sex, and APACHE II score.

**Fig 3:**
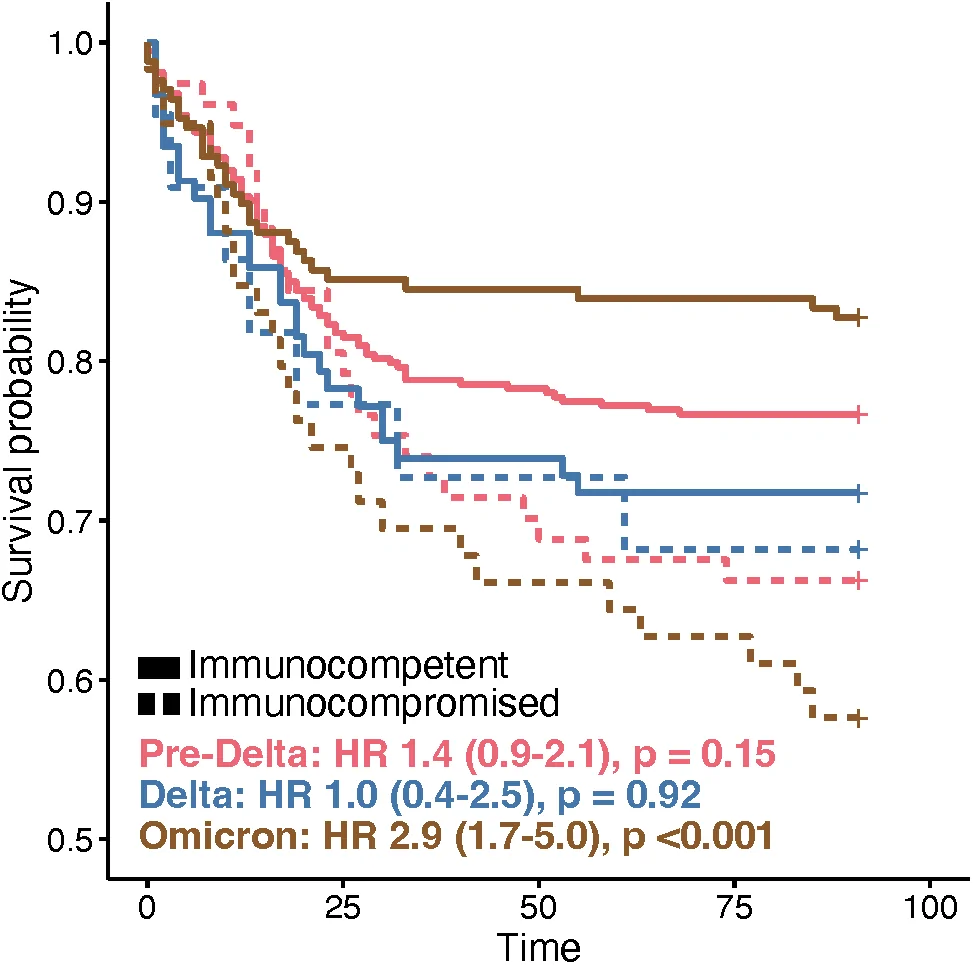
Kaplan-Meier survival curves evaluating 90-day survival in immunocompromised versus immunocompetent patients admitted to the ICU with SARS-CoV-2 infection separated by strain (Pre-Delta, Delta, and Omicron). Hazard ratio (HR) represents cox-proportion hazards ratio for 90-day survival in immunocompromised patients relative to immunocompetent adjusted for age, sex, and APACHE II score.

When analyzing the entire cohort, immunocompromise was associated with worse 90-day survival after adjustment for age, sex, and baseline APACHE II score (HR 1.68, 95% CI 1.2–2.3, p < 0.001, Fig 2). When stratified by strain period, this finding was driven by differences in outcomes during the Omicron period (Fig 3). 90-day mortality among immunocompromised patients admitted to the ICU during the Omicron era was 42%, compared to 17% mortality in immunocompetent patients (HR 2.9, 95% CI% 1.7–5.0, p < 0.001). In contrast, there was no statistically significant difference in 90-day mortality in immunocompromised patients relative to immunocompetent patients in either the pre-Delta period (34% versus 23%, p = 0.15) or the Delta period (32% versus 28%, p = 0.92) after adjustment for age, sex, and baseline APACHE II score. Kaplan-Meier survival analyses stratified by strain period are shown in Supplemental Figure 1 in S1 File. These 90-day mortality trends were largely consistent regardless of etiology of immunocompromise (Supplemental Table 3 in S1 File). Results were similar in our sensitivity analysis, including only patients admitted to ICU requiring high flow nasal cannula, non-invasive positive pressure ventilation, or mechanical ventilation (N = 518, including 104 immunocompromised subjects) Supplemental Figures 2, 3 in S1 File). Findings were also robust to additional adjustment for the Charlson Comorbidity Index; immunocompromised patients carried a higher comorbidity burden (median Charlson 3 [IQR 2–5] versus 1 [0–3]), yet immunocompromised status remained associated with higher 90-day mortality both overall (adjusted HR 1.55, 95% CI 1.12–2.15) and during the Omicron period (adjusted HR 2.74, 95% CI 1.57–4.79) (Supplemental Table 4 in S1 File).

### Differences in outcomes in immunocompromised patients were driven by improved outcomes in immunocompetent patients during Omicron

**Fig 4:**
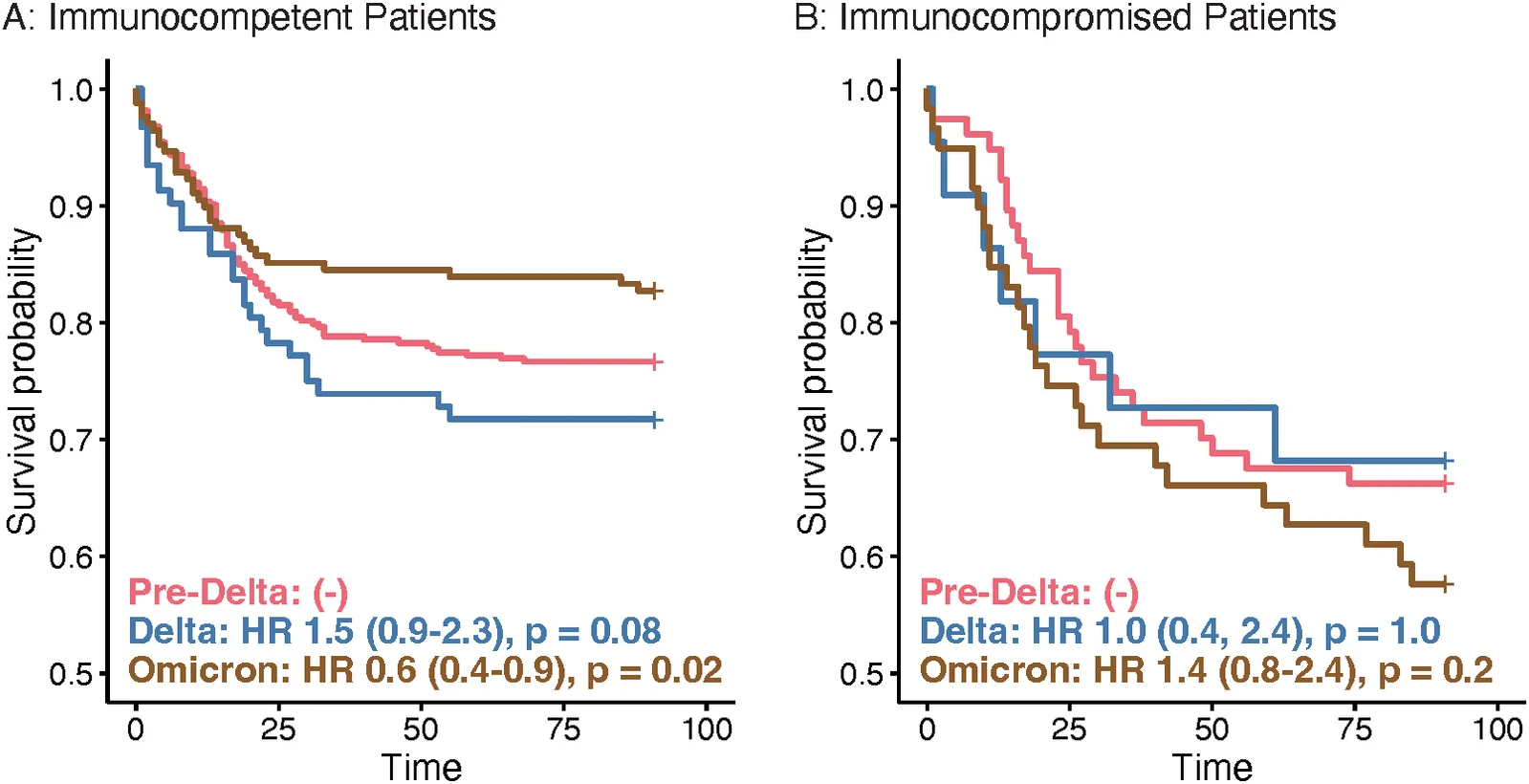
Kaplan-Meier survival curves evaluating 90-day survival in SARS-CoV-2 patients admitted to the ICU separated by immunocompromised status and strain (Pre-Delta, Delta, Omicron). Hazard ratio (HR) represents cox-proportion hazards ratio for 90-day survival in immunocompromised patients relative to immunocompetent adjusted for age, sex, and APACHE II score.

There were no statistically significant differences in 90-day survival among immunocompromised patients across variant periods, with survival rates of 66%, 68%, and 58% for pre-Delta, Delta, and Omicron, respectively (p = 0.96 for Delta, p = 0.24 for Omicron, compared to pre-Delta). There was, however, a striking difference in survival by strain period among immunocompetent patients. Immunocompetent patients with ICU admission during Omicron had an 83% 90-day survival rate compared to 77% during the pre-Delta and 72% during the Delta periods. Infection during the Omicron period was associated with significantly improved survival relative to Pre-Delta (HR 0.6, 95% CI 0.4–0.9, p = 0.02) (Fig 4A). Together, these findings suggest that the difference in outcomes between immunocompromised and immunocompetent patients across the variant periods was driven by improvement in outcomes in immunocompetent, but not immunocompromised, patients during Omicron.

### Vaccination status

We next evaluated whether differences in vaccination status across strain periods and by immunocompromised status might explain the observed disparities in outcomes. A total of 206 patients (26%) were documented as vaccinated in the Stanford EMR. As expected based on the timing of vaccine availability, the proportion of vaccinated patients varied by strain period: the vaccination rate during the Omicron period was 61%, compared to just 7% during the pre-Delta period and 30% during the Delta period. Vaccination rates also varied by immune status, with 46% (73 patients) of immunocompromised patients and 21% (133) of immunocompetent patients vaccinated.

**Fig 5:**
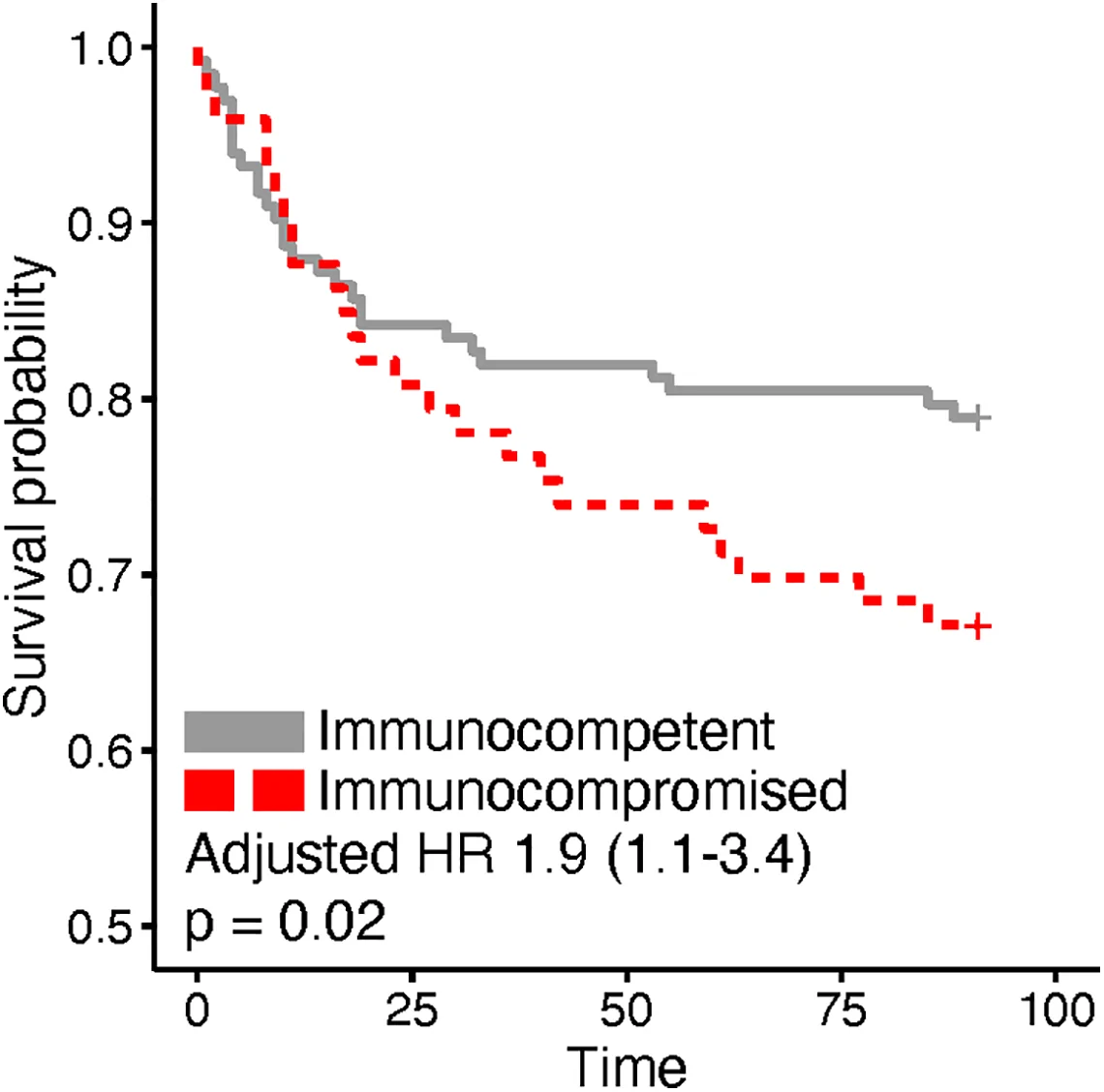
Kaplan-Meier survival curves evaluating 90-day survival in vaccinated immunocompromised versus vaccinated immunocompetent SARS-CoV-2 patients admitted to the ICU. Hazard ratio (HR) represents cox-proportion hazards ratio for 90-day survival in immunocompromised patients relative to immunocompetent adjusted for age, sex, and APACHE II score.

Outcomes were evaluated within the subset of vaccinated patients to assess whether differential immunity might account for the trends observed in the full cohort. In the 206 patients with reported vaccination, immunocompromised patients had worse outcomes, with a 90-day survival of 67% compared to 79% in immunocompetent patients (adjusted HR 1.9, 95% CI 1.1–3.4, p = 0.02, Fig 5), findings very consistent with the full cohort (Fig 2). Among vaccinated immunocompetent patients, survival was highest during the Omicron period, though differences across strain periods were not statistically significant. In contrast, vaccinated immunocompromised patients showed a significant difference in survival by strain period, with notably lower survival during Omicron (55% vs 88%, HR 4.4, 95% CI 1.0–18.6, p = 0.047, Supplemental Figure 4 in S1 File). However, interpretation of strain-specific comparisons is limited due to the small number of vaccinated immunocompromised patients during the pre-Delta (n = 15) and Delta (n = 11) periods.

## Discussion

In this large single health-care system cohort, immunocompromised patients who were admitted to the ICU with SARS-CoV-2 infection experienced higher mortality than immunocompetent patients. Notably, this difference in outcomes was predominantly driven by markedly improved survival among immunocompetent patients admitted during the Omicron-predominant period, while survival between the two groups was similar during pre-Delta and Delta periods. These findings suggest that the divergence in outcomes over time was driven primarily by improved survival among immunocompetent patients later in the pandemic. The exact etiology of this improvement in outcomes likely reflects numerous factors including recurrent exposure to COVID and/or vaccination reducing severity as well as the evolving treatment landscape during this time. This finding is hypothesis generating in terms of understanding the underlying pathogenesis of why survival improvements over the course of the pandemic lagged in immunocompromised vs immunocompetent patients. Our results echo previous cohort studies demonstrating persistent vulnerability among immunocompromised patients during the Omicron wave, even after receiving the primary set of COVID-19 vaccinations [9–12] The pronounced mortality difference during the Omicron period, despite the high proportion of vaccinated individuals, underscores the inadequate and incomplete immune protection that may follow vaccination or natural infection in immunocompromised patients  [18–23]. This apparent lack of improvement may reflect a combination of factors, including differences in immune protection from vaccination or prior infection, evolving treatment practices, possible strain-specific differences, and other unmeasured confounders. While Omicron is known to have increased transmissibility and altered host cell binding, the extent to which these viral characteristics contributed to the observed differences in outcomes among immunocompromised patients remains uncertain. This gap in knowledge is a significant barrier to the development of effective risk reduction strategies in this vulnerable patient group.

Our study has several strengths, including a large ICU cohort across multiple variant periods (pre-Delta, Delta, and Omicron) and inclusion of patients from both a tertiary referral and community hospital. Combining EMR-based data abstraction with manual phenotyping of immunocompromised status provided rich data to correct for comorbidities and illness severity. Sensitivity analyses limited to patients with known vaccination status further support the robustness of our findings.

There are several important limitations to our analysis worth noting. Our data extraction from the electronic STARR database limits the ability to exclude incidental COVID-19 infection, though our results were robust in a sensitivity analysis restricted to only patients who met respiratory failure criteria (requiring HFNC, BIPAP/CPAP, mechanical ventilation) at time of ICU admission. Vaccination status was only available for those with records linked to the Stanford Health System and likely underestimated true vaccination rates and did not account for booster doses or time since most recent vaccination. Additionally, we were underpowered to detect potential differences in the small number of patients who received vaccination in the pre-Delta and Delta periods. Nonetheless, among Omicron cases, approximately 60% of patients were documented as vaccinated, and mortality remained higher among immunocompromised patients in this subgroup.

our large overall cohort size, the number of immunocompromised patients within individual strain periods was relatively small, particularly during the Delta period. Consequently, estimates of mortality within these subgroups should be interpreted cautiously, as the study may have been underpowered to detect more subtle differences in outcomes across time periods. Furthermore, because multiple subgroup and sensitivity analyses were performed, findings from these exploratory analyses should be interpreted cautiously, particularly those with borderline statistical significance, as no formal correction for multiple testing was applied.

Because this study was conducted within a single healthcare system, our findings may not be fully generalizable to institutions with different patient demographics, healthcare access, vaccination uptake, ICU treatment protocols, or local viral epidemiology. Furthermore, viral strain periods were assigned based on local epidemiologic and genomic surveillance data rather than patient-level viral sequencing, introducing the potential for misclassification of strain, particularly near periods of variant transition. Finally, our study focused exclusively on ICU outcomes and therefore should not be generalized to immunocompromised populations outside the critical care setting. Our findings regarding mortality among vaccinated individuals should likewise not be extrapolated to non-ICU populations, where vaccination is known to protect against severe disease, even in immunocompromised patients.

## Conclusion

In this study, we found that immunocompromised patients admitted to the ICU with positive SARS-CoV-2 test experienced higher mortality than immunocompetent patients. This difference in outcomes differed by viral strain period and was largely driven by improved survival in immunocompetent patients in the Omicron period.

## Supporting information

S1 File This file contains Supplemental Tables 1–4 and Supplemental Figures 1–4. (DOCX)

## References

[1] Collins JP, Campbell AP, Openo K, Farley MM, Cummings CN, Hill M, et al. Outcomes of immunocompromised adults hospitalized with laboratory-confirmed influenza in the United States, 2011-2015. Clin Infect Dis. 2020;70(10):2121–30. doi: 10.1093/cid/ciz638 31298691 PMC7201407

[2] Dumas G, Bertrand M, Lemiale V, Canet E, Barbier F, Kouatchet A, et al. Prognosis of critically ill immunocompromised patients with virus-detected acute respiratory failure. Ann Intensive Care. 2023;13(1):101. doi: 10.1186/s13613-023-01196-9 37833435 PMC10575827

[3] Pereira MR, Mohan S, Cohen DJ, Husain SA, Dube GK, Ratner LE, et al. COVID-19 in solid organ transplant recipients: initial report from the US epicenter. Am J Transplant. 2020;20(7):1800–8. doi: 10.1111/ajt.15941 32330343 PMC7264777

[4] Krasnow MR, Litt HK, Lehmann CJ, Lio J, Zhu M, Sherer R. Cancer, transplant, and immunocompromising conditions were not significantly associated with severe illness or death in hospitalized COVID-19 patients. J Clin Virol. 2021;140:104850. doi: 10.1016/j.jcv.2021.104850 34022753 PMC8106963

[5] Goudsmit A, Cubilier E, Meert A-P, Aftimos P, Stathopoulos K, Spilleboudt C, et al. Factors associated with SARS-CoV-2 infection and outcome in patients with solid tumors or hematological malignancies: a single-center study. Support Care Cancer. 2021;29(11):6271–8. doi: 10.1007/s00520-021-06175-z 33851236 PMC8043836

[6] Sawyers A, Chou M, Johannet P, Gulati N, Qian Y, Zhong J, et al. Clinical outcomes in cancer patients with COVID-19. Cancer Rep (Hoboken). 2021;4(6):e1413. doi: 10.1002/cnr2.1413 34409775 PMC8420395

[7] Turtle L, Thorpe M, Drake TM, Swets M, Palmieri C, Russell CD, et al. Outcome of COVID-19 in hospitalised immunocompromised patients: an analysis of the WHO ISARIC CCP-UK prospective cohort study. PLoS Med. 2023;20(1):e1004086. doi: 10.1371/journal.pmed.1004086 36719907 PMC9928075

[8] Bytyci J, Ying Y, Lee LYW. Immunocompromised individuals are at increased risk of COVID-19 breakthrough infection, hospitalization, and death in the post-vaccination era: a systematic review. Immun Inflamm Dis. 2024;12(4):e1259. doi: 10.1002/iid3.1259 38661301 PMC11044684

[9] Bahremand T, Yao JA, Mill C, Piszczek J, Grant JM, Smolina K. COVID-19 hospitalisations in immunocompromised individuals in the Omicron era: a population-based observational study using surveillance data in British Columbia, Canada. Lancet Reg Health Am. 2023;20:100461. doi: 10.1016/j.lana.2023.100461 36890850 PMC9987330

[10] Singson JRC, Kirley PD, Pham H, Rothrock G, Armistead I, Meek J. Factors associated with severe outcomes among immunocompromised adults hospitalized for COVID-19—COVID-NET, 10 states, March 2020-February 2022. MMWR Morb Mortal Wkly Rep. 2022;71(27):878–84. doi: 10.15585/mmwr.mm7127a3 35797216 PMC9290380

[11] Belsky JA, Tullius BP, Lamb MG, Sayegh R, Stanek JR, Auletta JJ. COVID-19 in immunocompromised patients: a systematic review of cancer, hematopoietic cell and solid organ transplant patients. J Infect. 2021;82(3):329–38. doi: 10.1016/j.jinf.2021.01.022 33549624 PMC7859698

[12] Evans RA, Dube S, Lu Y, Yates M, Arnetorp S, Barnes E, et al. Impact of COVID-19 on immunocompromised populations during the Omicron era: insights from the observational population-based INFORM study. Lancet Reg Health Eur. 2023;35:100747. doi: 10.1016/j.lanepe.2023.100747 38115964 PMC10730312

[13] Arabi M, Al-Najjar Y, Mhaimeed N, Salameh MA, Paul P, AlAnni J, et al. Severity of the Omicron SARS-CoV-2 variant compared with the previous lineages: a systematic review. J Cell Mol Med. 2023;27(11):1443–64. doi: 10.1111/jcmm.17747 37203288 PMC10243162

[14] Lin L, Liu Y, Tang X, He D. The disease severity and clinical outcomes of the SARS-CoV-2 variants of concern. Front Public Health. 2021;9:775224. doi: 10.3389/fpubh.2021.775224 34917580 PMC8669511

[15] Sibai M, Wang H, Yeung PS-W, Sahoo MK, Solis D, Mfuh KO, et al. Development and evaluation of an RT-qPCR for the identification of the SARS-CoV-2 Omicron variant. J Clin Virol. 2022;148:105101. doi: 10.1016/j.jcv.2022.105101 35151048 PMC8816779

[16] Wang H, Miller JA, Verghese M, Sibai M, Solis D, Mfuh KO, et al. Multiplex SARS-CoV-2 genotyping reverse transcriptase PCR for population-level variant screening and epidemiologic surveillance. J Clin Microbiol. 2021;59(8):e0085921. doi: 10.1128/JCM.00859-21 34037430 PMC8373211

[17] Yeung PS-W, Wang H, Sibai M, Solis D, Yamamoto F, Iwai N, et al. Evaluation of a rapid and accessible reverse transcription-quantitative PCR approach for SARS-CoV-2 variant of concern identification. J Clin Microbiol. 2022;60(5):e0017822. doi: 10.1128/jcm.00178-22 35465708 PMC9119066

[18] Agrawal U, Bedston S, McCowan C, Oke J, Patterson L, Robertson C, et al. Severe COVID-19 outcomes after full vaccination of primary schedule and initial boosters: pooled analysis of national prospective cohort studies of 30 million individuals in England, Northern Ireland, Scotland, and Wales. Lancet. 2022;400(10360):1305–20. doi: 10.1016/S0140-6736(22)01656-7 36244382 PMC9560746

[19] Lee ARYB, Wong SY, Chai LYA, Lee SC, Lee MX, Muthiah MD, et al. Efficacy of COVID-19 vaccines in immunocompromised patients: systematic review and meta-analysis. BMJ. 2022;376:e068632. doi: 10.1136/bmj-2021-068632 35236664 PMC8889026

[20] Britton A, Embi PJ, Levy ME, Gaglani M, DeSilva MB, Dixon BE, et al. Effectiveness of COVID-19 mRNA vaccines against COVID-19-associated hospitalizations among immunocompromised adults during SARS-CoV-2 Omicron predominance—VISION Network, 10 states, December 2021-August 2022. MMWR Morb Mortal Wkly Rep. 2022;71(42):1335–42. doi: 10.15585/mmwr.mm7142a4 36264840 PMC9590295

[21] Duly K, Farraye FA, Bhat S. COVID-19 vaccine use in immunocompromised patients: a commentary on evidence and recommendations. Am J Health Syst Pharm. 2022;79(2):63–71. doi: 10.1093/ajhp/zxab344 34455440 PMC8499782

[22] Antinori A, Bausch-Jurken M. The burden of COVID-19 in the immunocompromised patient: implications for vaccination and needs for the future. J Infect Dis. 2023;228(Suppl 1):S4–12. doi: 10.1093/infdis/jiad181 37539764 PMC10401620

[23] Antinori A, Cicalini S, Meschi S, Bordoni V, Lorenzini P, Vergori A, et al. Humoral and Cellular immune response elicited by mRNA vaccination against severe acute respiratory syndrome coronavirus 2 (SARS-CoV-2) in people living with human immunodeficiency virus receiving antiretroviral therapy based on current CD4 T-Lymphocyte count. Clin Infect Dis. 2022;75(1):e552–63. doi: 10.1093/cid/ciac238 35366316 PMC9047161

