# Supplementary material for: Outcomes of immunocompromised and non-immunocompromised patients in the ICU diverge late in the pandemic
Source: PLoS One. 2026 Sep 9;21(9):e0357181. doi: 10.1371/journal.pone.0357181 (PMC13557382; doi:10.1371/journal.pone.0357181)
Supplement: Supplementary file 1 [file pone.0357181.s001.docx]

**Outcomes of immunocompromised and non-immunocompromised patients in the ICU diverge late in the pandemic**

Shreya Battu^1^, Andrew Moore^1^, Katie Lebold^2^, Anna Pacheco-Navarro^1^, Shaun Pienkos^1^, Christian O’Donnell^3^, Pablo Sanchez^4^, Jonasel Roque^1^, Caitlin Parmer-Chow^3^, Tara Ramaswamy^5^, Jenny Wilson^6^, Joe Levitt^1^, William Collins^7^, Angela J. Rogers^1*^

1. Stanford University School of Medicine, Division of Pulmonary, Allergy and Critical Care Medicine, Stanford, CA
2. Oregon Health & Sciences University, Division of Pulmonary, Allergy and Critical Care Medicine, Portland, OR
3. Stanford University School of Medicine, Department of Medicine, Stanford, CA
4. University of California San Francisco, Department of Medicine, San Francisco, CA
5. Stanford University, Department of Anesthesiology, Stanford, CA
6. Stanford University, Department of Emergency Medicine, Stanford, CA
7. Stanford University, Division of Hospital Medicine, Stanford, CA

*Corresponding Author:

Preferred Means of Contact: ajrogers@stanford.edu

Funding: Not applicable

**Supplemental Tables:**

**Supplemental Table 1:** Baseline demographics and clinical characteristics of ICU patients with SARS-CoV-2 infection by viral strain period. Values are presented as counts with percentages or medians with interquartile ranges, as appropriate.

|  | **Pre-Delta** | | **Delta** | **Omicron** | **All** |
| --- | --- | --- | --- | --- | --- |
| **Total** | | 450 | 114 | 227 | 791 |
| Age (median, IQR) | | 61 [45, 73] | 59 [43, 68] | 64 [50.5, 74.0] | 61 [46,73] |
| Gender (n, % female) | | 187 (42%) | 59 (52%) | 94 (41%) | 340 (43%) |
| **Race/Ethnicity** | | **-** | **-** | **-** | **-** |
| Asian/Pacific Islander | | 77 (16.9%) | 16 (14.5%) | 38 (16.6%) | 131 (16.6%) |
| Black/Native American | | 26 (5.8%) | 9 (7.9%) | 12 (5.2%) | 47 (5.9%) |
| White | | 101 (22%) | 39 (34%) | 80 (35%) | 220 (28%) |
| Hispanic/Latino | | 205 (46%) | 30 (26%) | 67 (30%) | 302 (38%) |
| Other/Unknown | | 41 (9.1%) | 20 (17.8%) | 30 (13.3%) | 91 (11.5%) |
| **Immunocompromised Status** | | 77 (17%) | 22 (19%) | 59 (26%) | 158 (20%) |
| Solid Organ Transplant | | 34 (7.7%) | 13 (11%) | 23 (10%) | 70 (9.0%) |
| BMT | | 4 (0.9%) | 0 (0%) | 2 (0.9%) | 6 (0.8%) |
| Heme malignancy | | 23 (5.2%) | 5 (4.4%) | 17 (7.5%) | 45 (5.8%) |
| Solid Malignancy on chemotherapy | | 10 (2.2%) | 1 (0.9%) | 7 (3.1%) | 18 (2.3%) |
| HIV/AIDS | | 4 (0.9%) | 0 (0%) | 0 (0%) | 4 (0.5%) |
| CTD | | 16 (3.6%) | 5 (4.4%) | 11 (4.9%) | 32 (4.1%) |
| **Treatments*** | |  |  |  |  |
| Steroids | | 367 (83%) | 91 (80%) | 148 (65%) | 606 (77%) |
| Remdesivir | | 287 (65%) | 63 (55%) | 87 (38%) | 437 (56%) |
| Tocilizumab | | 39 (8.8%) | 10 (8.8%) | 6 (2.6%) | 55 (7.0%) |
| Baricitinib | | 8 (1.8%) | 23 (20%) | 16 (7.0%) | 47 (6.0%) |
| Monoclonal Anti-spike Antibody | | 1 (0.2%) | 4 (3.5% | 0 (0%) | 5 (0.6) |
| **Markers of Severity** | |  |  |  |  |
| Shock | | 177 (39%) | 56 (49%) | 105 (46%) | 338 (43%) |
| APACHE (median, IQR) | | 16 [11, 23] | 17 [11.25, 23] | 17 [11.5, 22] | 16 [11, 23] |
| Intubated at Time of Admission (n, %) | | 161 (36%) | 41 (36%) | 70 (31%) | 272 (34%) |
| 90-Day Mortality (n, %) | | 113 (25%) | 33 (29%) | 54 (24%) | 200 (25%) |

*****Some therapies were given as part of interventional trials – in certain cases, treatment may have been placebo as opposed to treatment arm.

**Supplemental Table 2:** Multivariable logistic regression evaluating the odds of being immunocompromised across SARS-CoV-2 strain periods, adjusted for age and sex

| **Strain** | **Immunocompromised** | **Immunocompetent** | **Odds Ratio [CI]** | **p-value** |
| --- | --- | --- | --- | --- |
| Pre-Delta | 77 (49%) | 373 (59%) | -- | -- |
| Delta | 22 (14%) | 92 (14.5%) | 1.15 [0.67-1.93] | 0.6 |
| Omicron | 59 (37%) | 168 (26.5%) | 1.69 [1.15-2.49] | 0.008 |
| **Age** | 61 (median) | 61 (median) | 1.01 [1.0-1.02] | 0.3 |
| **Gender (male)** | 84 (53%) | 366 (58%) | 0.81 [0.57, 1.15] | 0.2 |

**Supplemental Table 3:** Mortality across immunocompromising condition and strain

| 90-Day Mortality Rate by Diagnosis and COVID-19 Strain | | | |
| --- | --- | --- | --- |
| Immunocompromising Diagnosis | Alpha | Delta | Omicron |
| Chemotherapy | 9/22 (40.9%) | 1/5 (20%) | 10/20 (50%) |
| Connective Tissue Disease | 9/16 (56.2%) | 3/5 (60%) | 3/11 (27.3%) |
| Hematologic malignancy | 7/23 (30.4%) | 2/5 (40%) | 9/17 (52.9%) |
| HIV/AIDS | 1/4 (25%) | NA | NA |
| Metastatic Malignancy | 6/10 (60%) | 0/2 (0%) | 6/8 (75%) |
| Solid Organ Transplant | 9/34 (26.5%) | 4/13 (30.8%) | 7/23 (30.4%) |

**Supplemental Table 4**: Sensitivity analysis of Cox proportional-hazards models for 90-day mortality with additional adjustment for the Charlson Comorbidity Index.

| **Analysis** | **N** | **Deaths** | **Primary model, HR (95% CI)** | **Charlson-adjusted, HR (95% CI)** |
| --- | --- | --- | --- | --- |
| ***Association of immunocompromised status with 90-day mortality (immunocompromised vs. immunocompetent)*** | | | | |
| Overall cohort | 791 | 200 | 1.69 (1.24–2.30), p<0.001 | 1.55 (1.12–2.15), p=0.01 |
| Pre-Delta period | 450 | 113 | 1.38 (0.89–2.14), p=0.15 | 1.18 (0.73–1.92), p=0.50 |
| Delta period | 114 | 33 | 1.05 (0.44–2.48), p=0.92 | 1.18 (0.47–2.95), p=0.73 |
| Omicron period | 227 | 54 | 2.97 (1.73–5.12), p<0.001 | 2.74 (1.57–4.79), p<0.001 |
| ***Association of the Omicron (vs. pre-Delta) period with 90-day mortality, within immune-status strata*** | | | | |
| Immunocompetent patients | 633 | 142 | 0.56 (0.36–0.85), p=0.01 | 0.55 (0.36–0.85), p=0.01 |
| Immunocompromised patients | 158 | 58 | 1.40 (0.80–2.44), p=0.24 | 1.49 (0.85–2.63), p=0.16 |
| ***Vaccinated cohort (immunocompromised vs. immunocompetent)*** | | | | |
| Immunocompromised status | 206 | 52 | 1.94 (1.11–3.39), p=0.02 | 1.83 (1.02–3.26), p=0.04 |

Primary models were adjusted for age, sex, and baseline APACHE II score; Charlson-adjusted models were additionally adjusted for the Charlson Comorbidity Index. Hazard ratios (HR) reflect the risk associated with immunocompromised status (vs. immunocompetent), except for the two Omicron-period rows, which reflect the risk of the Omicron period relative to the pre-Delta period within each immune-status stratum. CI, confidence interval.

**Supplemental Figures**

**Supplemental Figure 1:** Kaplan-Meier survival curves evaluating 90-day survival in immunocompromised versus immunocompetent SARS-CoV-2 patients admitted to the ICU separated by strain (Pre-Delta, Delta, and Omicron) Hazard ratio (HR) represents cox-proportion hazards ratio for 90-day survival in immunocompromised patients relative to immunocompetent adjusted for age, sex, and APACHE II score.

**Supplemental Figure 2:** Kaplan-Meier survival curves evaluating 90-day survival in immunocompromised versus immunocompetent SARS-CoV-2 patients with severe hypoxia admitted to the ICU. Hazard ratio (HR) represents cox-proportion hazards ratio for 90-day survival in immunocompromised patients relative to immunocompetent adjusted for age, sex, and APACHE II score.

**Supplemental Figure 3:** Kaplan-Meier survival curves evaluating 90-day survival in immunocompromised versus immunocompetent SARS-CoV-2 patients with severe hypoxia admitted to the ICU separated by strain (Pre-Delta, Delta, and Omicron). Hazard ratio (HR) represents cox-proportion hazards ratio for 90-day survival in immunocompromised patients relative to immunocompetent adjusted for age, sex, and APACHE II score.

**Supplemental Figure 4:** Kaplan-Meier survival curves evaluating 90-day survival in vaccinated immunocompromised versus vaccinated immunocompetent SARS-CoV-2 patients admitted to the ICU separated by strain (Pre-Delta, Delta, and Omicron). Hazard ratio (HR) represents cox-proportion hazards ratio for 90-day survival in immunocompromised patients relative to immunocompetent adjusted for age, sex, and APACHE II score.
