# Supplementary material for: Outcomes of immunocompromised and non-immunocompromised patients in the ICU diverge late in the pandemic
Source: PLoS One. 2026 Sep 9;21(9):e0357181. doi: 10.1371/journal.pone.0357181 (PMC13557382; doi:10.1371/journal.pone.0357181)
Supplement: Supplementary file 2 [file pone.0357181.s003.docx]

**AE Comments**

- *The manuscript addresses an important clinical topic. However, the reviewers have raised several substantive concerns regarding the methodology, interpretation of results, and presentation of the data. The authors are requested to provide a detailed point-by-point response to all reviewer comments and revise the manuscript accordingly. Based on the reviewers' evaluations, I recommend Major Revision.*

**We sincerely thank the editor and reviewers for their time and thoughtful comments. Based on their input, we have substantially revised the manuscript. We have added an additional sensitivity analysis to ensure the robustness of our findings after adjusting for comorbidity burden and have added COVID-directed therapies to supplemental table 1. In addition, we have thoroughly highlighted the important limitations of this EHR-based study to place the results in appropriate context. Please see our point-by-point response to reviewer comments below.**

***Comments to the Author*** *1. Is the manuscript technically sound, and do the data support the conclusions?

The manuscript must describe a technically sound piece of scientific research with data that supports the conclusions. Experiments must have been conducted rigorously, with appropriate controls, replication, and sample sizes. The conclusions must be drawn appropriately based on the data presented.*

*Reviewer #1: Yes*

*Reviewer #2: Yes*

*2. Has the statistical analysis been performed appropriately and rigorously?*

*Reviewer #1: Yes*

*Reviewer #2: Yes*

*3. Have the authors made all data underlying the findings in their manuscript fully available?

The* [*PLOS Data policy*](https://urldefense.com/v3/__http:/track.editorialmanager.com/CL0/http:*2F*2Fwww.plosone.org*2Fstatic*2Fpolicies.action*23sharing/1/010f019e90449f89-f8234817-8c4e-479b-966e-c5f0a90b3b1d-000000/MQmLbwV2_Hrjgm6dI-E5t5GxL_CNulAEZ7IVAgT2I5o=258__;JSUlJSU!!G92We9drHetJ8EofZw!c-GBRMJ7d11w9LQc5KK27KRRKa48wpoStdR1TLn3e7mFqJHfg55psuJhdMRjrM9Pi429Z_XX6jczBO7f9d0$) *requires authors to make all data underlying the findings described in their manuscript fully available without restriction, with rare exception (please refer to the Data Availability Statement in the manuscript PDF file). The data should be provided as part of the manuscript or its supporting information, or deposited to a public repository. For example, in addition to summary statistics, the data points behind means, medians and variance measures should be available. If there are restrictions on publicly sharing data—e.g. participant privacy or use of data from a third party—those must be specified.*

*Reviewer #1: Yes*

*Reviewer #2: No*

**Thank you for this note. We have created a deidentified dataset and have included this CSV in the supplement.**

*4. Is the manuscript presented in an intelligible fashion and written in standard English?

PLOS ONE does not copyedit accepted manuscripts, so the language in submitted articles must be clear, correct, and unambiguous. Any typographical or grammatical errors should be corrected at revision, so please note any specific errors here.*

*Reviewer #1: Yes*

*Reviewer #2: Yes*

*5. Review Comments to the Author

Please use the space provided to explain your answers to the questions above. You may also include additional comments for the author, including concerns about dual publication, research ethics, or publication ethics. (Please upload your review as an attachment if it exceeds 20,000 characters)*

***Reviewer #1:*** *Manuscript ID: PONE-D-26-19619

The manuscript titled “Outcomes of immunocompromised and non-immunocompromised patients in the ICU diverge late in the pandemic” aimed to evaluate how immunocompromised status affected survival in critically ill COVID-19 patients during the pre-Delta, Delta, and Omicron periods. The results showed that immunocompromised patients had much higher mortality during the Omicron period, likely due to weaker protection from vaccination or prior infection compared to immunocompetent patients. Overall, the study is interesting; however, the authors are required to address the following comments before the manuscript can be considered for publication.*

**We sincerely thank you for your thoughtful comments and suggestions. We have carefully considered each point and revised the manuscript accordingly. Our detailed responses are provided below.**

*1.A major unaddressed confounder is the evolution of COVID-19 treatment protocols in ICU during the study period (2020–2022). Various trial showing benefits of anti-inflammatory, monoclonal antibodies and antiviral such as Dexamethasone became standard of care after the RECOVERY trial (mid 2020), tocilizumab was adopted in 2021. Improved outcomes during Omicron may reflect advances in the above therapies, in addition to vaccination and prior immunity. The manuscript attributes outcome differences mainly to impaired vaccine response without adjusting for treatment variation. At a minimum, a table describing treatment utilization (corticosteroids, remdesivir, tocilizumab, monoclonal antibodies, antivirals such as nirmatrelvir/ritonavir) by period and immune status is needed.*

**We thank the reviewer for this important suggestion and agree that changes in COVID-19 treatment practices may have contributed to improvements in outcomes during later stages of the pandemic which particularly improved outcomes in immunocompetent patients. Importantly, Stanford was not particularly hard-hit with COVID-19 in the March-June 2020 time frame prior to the publication of the RECOVER trial. As a result, even in the pre-Delta period, the vast majority of patients received routine anti-viral and steroid therapy. To address this concern, we have added a supplemental table summarizing the utilization of major COVID-19 therapies, including corticosteroids, remdesivir, tocilizumab, and monoclonal antibodies, stratified by viral strain (Supplemental table 1).**

|  | **Pre-Delta** | | **Delta** | **Omicron** | **All** |
| --- | --- | --- | --- | --- | --- |
| **Total** | | 450 | 114 | 227 | 791 |
| **Treatments*** | |  |  |  |  |
| Steroids | | 367 (83%) | 91 (80%) | 148 (65%) | 606 (77%) |
| Remdesivir | | 287 (65%) | 63 (55%) | 87 (38%) | 437 (56%) |
| Tocilizumab | | 39 (8.8%) | 10 (8.8%) | 6 (2.6%) | 55 (7.0%) |
| Baricitinib | | 8 (1.8%) | 23 (20%) | 16 (7.0%) | 47 (6.0%) |
| Monoclonal Anti-spike Antibody | | 1 (0.2%) | 4 (3.5% | 0 (0%) | 5 (0.6) |

**We have expanded the Discussion to acknowledge that evolving treatment protocols likely contributed to the improved survival observed in immunocompetent patients later in the pandemic, a benefit that was not seen in immunocompromised patients to the same extent. This finding further supports our primary conclusion that immunocompromised patients did not experience the same survival gains later in the pandemic, despite increasing availability of both effective therapies and population-level immunity.**

**The following text has been added to the Discussion:**

" These findings suggest that the divergence in outcomes over time was driven primarily by improved survival among immunocompetent patients later in the pandemic. The exact etiology of this improvement in outcomes likely reflects numerous factors including recurrent exposure to COVID and/or vaccination reducing severity as well as the evolving treatment landscape during this time. This finding is hypothesis generating in terms of understanding the underlying pathogenesis of why survival improvements over the course of the pandemic lagged in immunocompromised vs immunocompetent patients.”

*2.The Cox models adjust only for age, sex, and APACHE II score. Important confounders such as BMI/obesity, diabetes, chronic kidney disease, chronic lung disease, and other comorbidities are not included, which are largely factors in immunocompromised patients. Given that comorbidity profiles may differ between immunocompromised and immunocompetent patients and across time periods, residual confounding is a concern. Larger studies in this space have adjusted for comorbidity burden using indices such as the Charlson Comorbidity Index.*

**We appreciate this comment. We selected APACHE II because it captures both acute illness severity and several chronic health conditions, including chronic organ dysfunction and immunocompromised status. Additionally, one of the strengths of our study is the use of manual phenotyping of immunocompromised status through detailed chart review rather than reliance solely on administrative codes.**

**To further address the reviewer's concern regarding residual confounding, we performed an additional sensitivity analysis adjusting for major comorbidities, including the Charlson Comorbidity Index. The results were essentially unchanged: immunocompromised status remained independently associated with 90-day mortality in the overall cohort (adjusted HR 1.55, 95% CI 1.12–2.15) and during the Omicron period (adjusted HR 2.74, 95% CI 1.57–4.79), and the improved survival among immunocompetent patients during the Omicron period persisted (adjusted HR 0.55, 95% CI 0.36–0.85) (Supplemental Table 4), supporting the robustness of our findings. These findings have been added to our results as below:**

**“**Findings were also robust to additional adjustment for the Charlson Comorbidity Index; immunocompromised patients carried a higher comorbidity burden (median Charlson 3 [IQR 2–5] versus 1 [0–3]), yet immunocompromised status remained associated with higher 90-day mortality both overall (adjusted HR 1.55, 95% CI 1.12–2.15) and during the Omicron period (adjusted HR 2.74, 95% CI 1.57–4.79) (**Supplemental Table 4**).”

**We have included supplemental table 4 below for reference**

Supplemental Table 4: Sensitivity analysis of Cox proportional-hazards models for 90-day mortality with additional adjustment for the Charlson Comorbidity Index.

| **Analysis** | **N** | **Deaths** | **Primary model, HR (95% CI)** | **Charlson-adjusted, HR (95% CI)** |
| --- | --- | --- | --- | --- |
| ***Association of immunocompromised status with 90-day mortality (immunocompromised vs. immunocompetent)*** | | | | |
| Overall cohort | 791 | 200 | 1.69 (1.24–2.30), p<0.001 | 1.55 (1.12–2.15), p=0.01 |
| Pre-Delta period | 450 | 113 | 1.38 (0.89–2.14), p=0.15 | 1.18 (0.73–1.92), p=0.50 |
| Delta period | 114 | 33 | 1.05 (0.44–2.48), p=0.92 | 1.18 (0.47–2.95), p=0.73 |
| Omicron period | 227 | 54 | 2.97 (1.73–5.12), p<0.001 | 2.74 (1.57–4.79), p<0.001 |
| ***Association of the Omicron (vs. pre-Delta) period with 90-day mortality, within immune-status strata*** | | | | |
| Immunocompetent patients | 633 | 142 | 0.56 (0.36–0.85), p=0.01 | 0.55 (0.36–0.85), p=0.01 |
| Immunocompromised patients | 158 | 58 | 1.40 (0.80–2.44), p=0.24 | 1.49 (0.85–2.63), p=0.16 |
| ***Vaccinated cohort (immunocompromised vs. immunocompetent)*** | | | | |
| Immunocompromised status | 206 | 52 | 1.94 (1.11–3.39), p=0.02 | 1.83 (1.02–3.26), p=0.04 |

Primary models were adjusted for age, sex, and baseline APACHE II score; Charlson-adjusted models were additionally adjusted for the Charlson Comorbidity Index. Hazard ratios (HR) reflect the risk associated with immunocompromised status (vs. immunocompetent), except for the two Omicron-period rows, which reflect the risk of the Omicron period relative to the pre-Delta period within each immune-status stratum. CI, confidence interval.

*3.The definition of fully vaccinated patients as described by the author (1dose J&J or 2 doses Moderna or Pfizer vaccines) does not address booster doses, which were widely available during the Omicron period and are known to provide additional protection, particularly in immunocompromised patients. This omission limits the ability to assess the role of vaccination adequacy.*

**Thank you for highlighting this limitation. We agree that booster doses likely conferred additional protection, particularly among immunocompromised patients. Unfortunately, complete booster information was not consistently available for all patients and therefore could not be reliably incorporated into our analyses.**

**We have now explicitly acknowledged this limitation in the Discussion:**

“Vaccination status was only available for those with records linked to the Stanford Health System and likely underestimated true vaccination rates and did not account for booster doses or time since most recent vaccination. Additionally, we were underpowered to detect potential differences in the small number of patients who received vaccination in the pre-Delta and Delta periods.”

*4.Multiple stratified analyses are performed (overall, by strain, by vaccination status, and sensitivity analyses) without correction for multiple testing. While this is common in exploratory analyses, the authors should acknowledge the risk of type I error, particularly for borderline-significant findings (e.g., HR 4.4, 95% CI 1.0–18.6, p=0.047 for vaccinated immunocompromised patients during Omicron).*

**We appreciate this comment and agree that multiple exploratory analyses increase the risk of type I error, particularly for borderline-significant findings.**

**We have added the following statement to the Limitations section:**

“Because multiple subgroup and sensitivity analyses were performed, findings from exploratory analyses should be interpreted cautiously, particularly those with borderline statistical significance, as no formal correction for multiple testing was applied”.

*5.Supplemental Table 2 is described as "Logistic regression evaluating the association between immunocompromised status and 90-day mortality across viral strain periods" in the table title, but the text describes it as evaluating the association of strain with immunocompromised status (i.e., the outcome is immunocompromised status, not mortality). The table content (showing odds of being immunocompromised by strain) matches the text description, but the table title is incorrect and should be revised.*

**Thank you for identifying this error. We have corrected the title of Supplemental Table 2 to accurately reflect the analysis performed:**

"Multivariable logistic regression evaluating the odds of being immunocompromised across SARS-CoV-2 strain periods, adjusted for age and sex."

*6.Line 64: "stains" should be "strains."*

**Thank you for identifying this typographical error. This has been corrected in the revised manuscript.** *7.Table 1: The "All" column for Black/Native American shows 4.1%, but should be approximately 5.9% based on 47/791*

**Thank you for bringing this to our attention. We have corrected the percentage in Table 1.**

*8.The abstract lists the corresponding author as "Shreya Battu, BS" while the manuscript body lists "Angela J. Rogers, MD, MPH" as the corresponding author; this discrepancy should be resolved.*

**Thank you for identifying this inconsistency. The corresponding author information has been corrected throughout the manuscript to consistently identify Dr. Angela J. Rogers as the corresponding author.**

*9.Figures should be provided in high resolution to ensure clarity and readability.*

**Thank you for this suggestion. All figures have been regenerated and uploaded as high-resolution TIFF files in accordance with the journal's figure requirements.**

*10.Grammar should be carefully checked and corrected throughout the manuscript.*

**Thank you for this recommendation. The manuscript has undergone a thorough review for grammar, spelling, and readability, and several minor edits have been made throughout.**
 *11.References should be verified and formatted consistently throughout the manuscript according to the journal’s guidelines.*

**Thank you for this comment. We have carefully reviewed all references and revised them to ensure consistency and compliance with the journal's formatting requirements.**

***Reviewer #2****: The manuscript by Butta et al., entitled outcomes of immunocompromised and non-immunocompromised patients in the ICU diverge late in the pandemic,” is a well-designed retrospective cohort study. This manuscript addresses a relevant question of how the risk profile for immunocompromised patients shifted across SARS-CoV-2 variants. However, the manuscript has several data gaps and sample sizes that must be addressed before publication.*

**We sincerely thank the reviewer for their thoughtful and constructive comments. We appreciate the positive assessment of our study design and have substantially revised the manuscript to address the concerns raised. Our detailed responses are provided below.**

*1. The authors demonstrate a robust dataset of 791 ICU patients over two years, which signifies an important clinical paradox that although the omicron variant coincided with lower mortality for immunocompromised patients, but it is also lethal for the immunocompromised populations.*

**We thank the reviewer for this supportive comment and agree that the persistent vulnerability of immunocompromised patients despite improving outcomes in the broader population represents an important and clinically relevant finding.**

*2. While the author demonstrates the robust overall sample size for immunocompromised subgroups during the Delta (n=22) and Omicron (n=59) are relatively small. Even the lack of statistical significance in the Delta period may simply be a reflection of an underpowered subgroup rather than a true biological similarity in mortality rates. The author must explicitly discuss the limitations of these small subgroup sizes in the discussion section.*

**We agree with the reviewer that the relatively small number of immunocompromised patients within individual strain periods limits the precision of our subgroup analyses and the strength of conclusions that can be drawn regarding temporal differences in outcomes.**

**We have expanded the Limitations section to emphasize this point:**

“Despite our large overall cohort size, the number of immunocompromised patients within individual strain periods was relatively small, particularly during the Delta period. Consequently, estimates of mortality within these subgroups should be interpreted cautiously, as the study may have been underpowered to detect subtle differences in outcomes across time periods. Furthermore, because multiple subgroup and sensitivity analyses were performed, findings from these exploratory analyses should be interpreted cautiously, particularly those with borderline statistical significance, as no formal correction for multiple testing was applied.”

*3. The study is conducted entirely at Stanford, and the findings reflect the demographic, high vaccination rates, and the specific ICU protocols of the medical center. The authors need to address how the institutional biases might limit the findings to community hospital settings with different baseline healthcare access.*

**We appreciate this important point regarding generalizability. Our study was conducted within a single healthcare system, which includes both a tertiary academic medical center and a community affiliate hospital. Nevertheless, the patient population, treatment protocols, and vaccination uptake within our institution may differ from those of other healthcare settings.**

**We have added the following statement to the Discussion:**

"Because this study was conducted within a single healthcare system, our findings may not be fully generalizable to institutions with different patient demographics, healthcare access, vaccination uptake, or ICU treatment protocols."

*4. The authors must provide the methodologies that describes theee classifications of variants as strictly on date ranges rather than actual viral genotyping or sequencing data. The authors should clarify if any sequencing data were available to validate these time period boundaries or list this as a limitation in the manuscript.*

**We thank the reviewer for this suggestion and have clarified our methodology. Viral strain periods were defined using institutional genomic surveillance data demonstrating sequential predominance of specific variants within the Stanford Health Care population rather than patient-level sequencing data.**

**The Methods section has been revised to state:**

"COVID-19 strain periods were assigned based on local epidemiologic and institutional genomic surveillance data demonstrating distinct periods of variant predominance within the Stanford Health Care population, rather than patient-level viral sequencing, as this data was not available.”

**We have also added the following statement to the Limitations section:**

"Furthermore, viral strain periods were assigned based on local epidemiologic and genomic surveillance data rather than patient-level viral sequencing, introducing the potential for misclassification of strain, particularly near periods of variant transition”

*5. The authors should include a table breaking down the 90-day mortality by specific condition types (e.g., did solid organ transplant patients fare worse than those with connective tissue disease during Omicron?) in the results section.*

**Thank you for this suggestion. Overall, because the n was quite small, we felt underpowered to make meaningful evaluations across individual causes of immunosuppression, though we agree that this will be of interest to readership. We have added a Supplemental table (Supplemental Table 3) to describe these outcomes without statistics and have included this below for convenience.**

| 90-Day Mortality Rate by Diagnosis and COVID-19 Strain | | | |
| --- | --- | --- | --- |
| Immunocompromising Diagnosis | Alpha | Delta | Omicron |
| Chemotherapy | 9/22 (40.9%) | 1/5 (20%) | 10/20 (50%) |
| Connective Tissue Disease | 9/16 (56.2%) | 3/5 (60%) | 3/11 (27.3%) |
| Hematologic malignancy | 7/23 (30.4%) | 2/5 (40%) | 9/17 (52.9%) |
| HIV/AIDS | 1/4 (25%) | NA | NA |
| Metastatic Malignancy | 6/10 (60%) | 0/2 (0%) | 6/8 (75%) |
| Solid Organ Transplant | 9/34 (26.5%) | 4/13 (30.8%) | 7/23 (30.4%) |

*6. The authors have indicated that the widening mortality gap during the Omicron era is due to an “impaired immune response to vaccination.” However, individual vaccination data (e.g., number of doses, time since last dose) and the administration of variant-specific therapeutics such as monoclonal antibodies Paxlovid and Remdesivir are not detailed in the methods or in the results. The treatment guidelines and vaccine availability shifted drastically between March 202 and July 2022. The authors must include either a control for vaccination/treatment status in their model or explicitly mention it in their conclusions to reflect the missing data.*

**We appreciate this important comment. We agree that our observational data does not allow us to definitely attribute the widening mortality gap during the Omicron period solely to impaired vaccine responses. We have removed this statement from our conclusion and we have tempered our language throughout the manuscript. We have added the following sentence in our discussion**

“This apparent lack of improvement may reflect a combination of factors, including differences in immune protection from vaccination or prior infection, evolving treatment practices, possible strain-specific differences, and other unmeasured confounders”

**Additionally, we performed sensitivity analyses restricted to patients with known vaccination status, which demonstrated findings consistent with the primary analysis. We have expanded our Discussion to acknowledge that information regarding booster doses, timing of vaccination, and certain outpatient therapies was incomplete and therefore could not be fully incorporated into our models.**

" These findings suggest that the divergence in outcomes over time was driven primarily by improved survival among immunocompetent patients later in the pandemic. The exact etiology of this improvement in outcomes likely reflects numerous factors including recurrent exposure to COVID and/or vaccination reducing severity as well as the evolving treatment landscape during this time. This finding is hypothesis generating in terms of understanding the underlying pathogenesis of why survival improvements over the course of the pandemic lagged in immunocompromised vs immunocompetent patients.”

“Vaccination status was only available for those with records linked to the Stanford Health System and likely underestimated true vaccination rates and did not account for booster doses or time since most recent vaccination. Additionally, we were underpowered to detect potential differences in the small number of patients who received vaccination in the pre-Delta and Delta periods.”

**Finally, we note that incomplete ascertainment of vaccination status would likely bias our findings toward the null, as immunocompromised patients may have been more likely to receive vaccination than immunocompetent patients.**
